# Exosome-Related Multi-Pass Transmembrane Protein TSAP6 Is a Target of Rhomboid Protease RHBDD1-Induced Proteolysis

**DOI:** 10.1371/journal.pone.0037452

**Published:** 2012-05-18

**Authors:** Chunhua Wan, Jun Fu, Yong Wang, Shiying Miao, Wei Song, Linfang Wang

**Affiliations:** 1 National Laboratory of Medical Molecular Biology, Institute of Basic Medical Sciences, Chinese Academy of Medical Sciences, Peking Union Medical College, China; 2 School of Public Health, Nantong University, Nantong, China; University of Rome, Italy

## Abstract

We have previously reported that rhomboid domain containing 1 (RHBDD1), a mammalian rhomboid protease highly expressed in the testis, can cleave the Bcl-2 protein Bik. In this study, we identified a multi-pass transmembrane protein, tumor suppressor activated pathway-6 (TSAP6) as a potential substrate of RHBDD1. RHBDD1 was found to induce the proteolysis of TSAP6 in a dose- and activity-dependent manner. The cleavage of TSAP6 was not restricted to its glycosylated form and occurred in three different regions. In addition, mass spectrometry and mutagenesis analyses both indicated that the major cleavage site laid in the C-terminal of the third transmembrane domain of TSAP6. A somatic cell knock-in approach was used to genetically inactivate the endogenous RHBDD1 in HCT116 and RKO colon cancer cells. Exosome secretion was significantly elevated when RHBDD1 was inactivated in the two cells lines. The increased exosome secretion was verfied through the detection of certain exosomal components, including Tsg101, Tf-R, FasL and Trail. In addition, the elevation of exosome secretion by RHBDD1 inactivation was reduced when TSAP6 was knocked down, indicating that the role of RHBDD1 in regulating exosomal trafficking is very likely to be TSAP6-dependent. We found that the increase in FasL and Trail increased exosome-induced apoptosis in Jurkat cells. Taken together, our findings suggest that RHBDD1 is involved in the regulation of a nonclassical exosomal secretion pathway through the restriction of TSAP6.

## Introduction

Over the past few decades, studies have found that regulated intramembrane proteolysis (RIP) plays an important role in various kinds of cellular processes, including cell signaling, gene transcription and apoptosis [1], [2]. Regulated intramembrane proteolysis is a process through which proteases cleave substrates within their transmembrane regions. Three major intramembrane protease families have been identified, including the metalloprotease-type S2P family, the γ-secretase and signal peptide peptidase family and the rhomboid proteases.

Rhomboid proteins are a family of widely conserved intramembrane serine proteases found from bacteria to humans [3]. In Drosophila, they cleave the epidermal growth factor-like ligands to regulate the EGFR signaling pathway, playing important roles in signal transduction pathways and organism development [4], [5]. The mitochondrial rhomboid protease PARL has been found to regulate mitochondrial membrane remodeling and apoptosis [6], [7]. Rhomboid proteases are also involved in para-invasion and replication [8]. Although significant progress has been made since their discovery approximately ten years ago, the biological functions of most rhomboid proteases remain unclear. Few substrates have been identified for the rhomboid proteases, and the identification of these substrates will play a key role in understanding the function of this intriguing family of intramembrane proteases. Thus, finding novel substrates is an urgent need for the investigation of rhomboid proteases. Furthermore, only single transmembrane proteins have been identified as substrates of rhomboid proteases [9], [10], [11]. However, because so many transmembrane proteins are multi-pass membrane proteins, there is a question regarding whether any multi-pass transmembrane proteins are substrates for rhomboid proteases.

TSAP6/STEAP3 was initially identified as a p53-inducible gene implicated in apoptosis and cell cycle regulation [12], [13]. TSAP6 is a member of the six-transmembrane epithelial antigen of the prostate (STEAP) family and facilitates ferrireductase iron uptake in erythroid cells [14], [15]. However, the most intriguing finding is that TSAP6 plays a role in the regulation of nonclassical exosomal secretion, which may have a broad biological significance [16], [17].

Exosomes, which are 30–100 nm in diameter, are derived from the internal vesicles of multivesicular bodies. They are secreted by various types of cells through a nonclassical secretion pathway [18], [19]. Recent studies have highlighted the role of exosomes in various kinds of cellular processes, including cell maturation and differentiation, cancer progression and immune regulation [20], [21]. The function of the exosome is diverse and complicated and is related to its origin, its numerous components, and its targeted cells [22], [23], [24], [25]. However, the mechanism by which exosome secretion is regulated is still unclear. Exosomes originate from intracellular membrane vesicles, such as endosomes, lysosomes, and the plasma membrane, and they are secreted through fusion with the plasma membrane [26], [27]. Studies have found that various kinds of factors and proteins can influence exosome secretion, including calcium, DGKα, Rab family proteins and p53 [28], [29], [30], [31]. Interestingly, p53 has been found to promote exosome secretion through up-regulating TSAP6 transcription [16].

We investigated the function of the mammalian rhomboid protein RHBDD1 and found that it could promote the proteolytic cleavage and subsequent proteasomal degradation of the pro-apoptotic Bcl-2 family member Bik [32]. We also found RHBDD1 to be implicated in the regulation of spermatogenesis [33]. Bik was found to interact with RHBDD1 using a yeast two-hybrid screen with RHBDD1 as bait. In addition to Bik, the multi-transmembrane protein TSAP6 was also identified in the screen. In this study, we investigated whether TSAP6 was a novel substrate of RHBDD1. We found that RHBDD1 induced the cleavage of multi-transmembrane protein TSAP6. The major cleavage site was mapped through mass spectrometry and mutagenesis experiments. We also show that the level of TSAP6-mediated exosomal secretion was elevated when RHBDD1 was mutated. Our findings may help clarify the mechanism by which rhomboid proteases are involved in the cellular secretion pathway in mammals.

## Results

### RHBDD1 overexpression induces the cleavage of TSAP6

**Figure 1:**
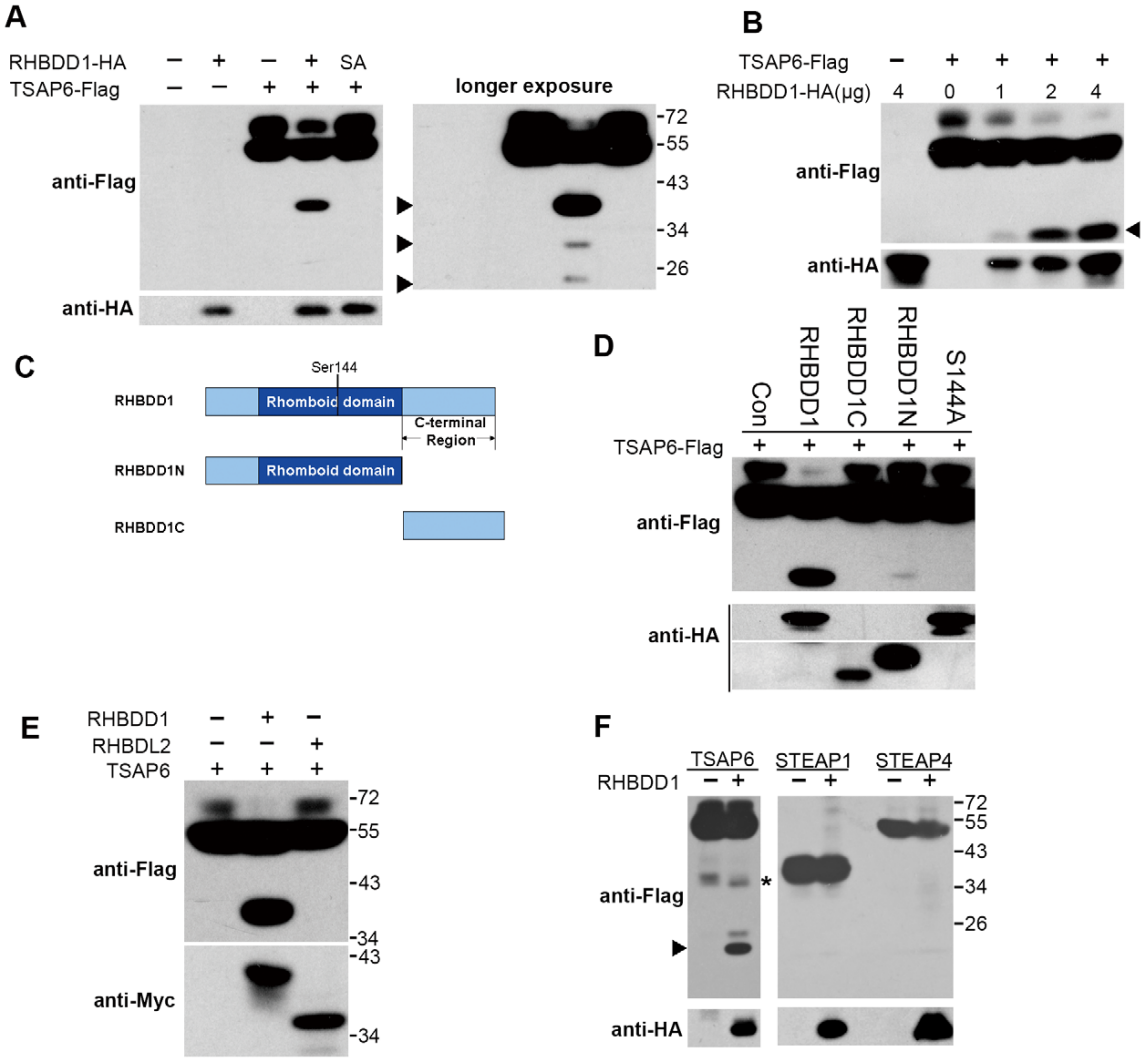
RHBDD1 overexpression induces the cleavage of TSAP6. A. RHBDD1 mediated proteolytic processing of TSAP6 in an enzymatic activity-dependent manner. Upon co-expression of TSAP6-Flag and RHBDD1-HA or RHBDD1^S144A^-HA in HEK-293T cells, the cleaved fragments were observed only with RHBDD1 overexpression. Note that three cleaved fragments (black arrowheads) were detected when the blot was exposed for a longer period of time. B. Dose dependency of RHBDD1-induced cleavage of TSAP6. The lysates of HEK-293T cells transfected with the indicated amounts of RHBDD1-HA and TSAP6-Flag constructs were blotted using anti-Flag and anti-HA antibodies. The cleaved fragments are indicated with black arrowheads. C. RHBDD1 and its truncated forms. D. Determination of the cleaving ability of different regions of RHBDD1 on TSAP6. The lysates of 293T cells transfected with TSAP6 and empty vector or RHBDD1-HA, RHBDD1C-HA, RHBDD1N-HA, and RHBDD1^S144A^-HA (here designated as S144A) were seperated with 10% SDS-PAGE and blotted with the indicated antibodies. E. RHBDL2 was not found to cleave TSAP6. F. RHBDD1 did not promote proteolysis of STEAP1 and STEAP4. 3xFlag-tagged TSAP6, STEAP1 or STEAP4 constructs were co-transfected with empty vectors or RHBDD1-HA. The cell lysates were blotted with indicated antibodies. The cleavage band of TSAP6 was shown with black arrowhead. A non-specific band was indicated with an asterisk.

Because RHBDD1 is a serine-type intramembrane protease, we hypothesize that transmembranal TSAP6 may be a substrate of RHBDD1. We co-transfected a Flag-tagged TSAP6 construct with RHBDD1 or with its catalytically inactive form, RHBDD1^S144A^ [32]. When transfected alone, Flag-tagged TSAP6 appeared to be an approximately 55–70 kDa doublet on the immunoblot (Fig. 1A), probably as a result of posttranscriptional glycosylation, as previously reported [17]. However, the pattern of the bands of TSAP6 was different when it was cotransfected with RHBDD1. The intensity of the 55–70 kDa TSAP6 doublet decreased dramatically, and a lower band (approximately 38 kDa) appeared (Fig. 1A). However, RHBDD1^S144A^ did not show this effect, indicating that the catalytic activity of RHBDD1 is essential for the cleavage of TSAP6. In addition to the 38-kDa cleavage band, two weaker cleavage bands were detected when the blot was exposed for a longer period of time (Fig. 1A). The two weaker cleavage bands were visible in most cases during our investigation of the cleavage when exposed for longer periods. To determine whether RHBDD1 overexpression was responsible for the cleavage of TSAP6, the effect of the level of RHBDD1 on cleavage was investigated. The proteolytic cleavage of TSAP6 increased gradually when the expression of RHBDD1 was increased (Fig. 1B). Different parts of RHBDD1 were tested for their ability to induce the cleavage of TSAP6. RHBDD1 is mainly composed of an N-terminal rhomboid domain and a C-terminal domain (Fig. 1C). The N-terminal region (1–214 aa, mainly composed of the 59–214 aa rhomboid domain) of RHBDD1 was sufficient for the proteolysis of TSAP6, but its activity was much weaker than full-length RHBDD1 (Fig. 1D).

The specificity of RHBDD1 for TSAP6 processing was also evaluated using another mammalian rhomboid protease, RHBDL2, for which several substrates have been identified [11], [34], [35]. TSAP6 was cotransfected with Myc-tagged RHBDD1 or RHBDL2 into HEK-293T cells to determine whether RHBDL2 overexpression might also result in TSAP6 cleavage (Fig. 1E). No cleaved fragments were detected when TSAP6 was co-expressed with RHBDL2, indicating that not all rhomboid proteases have the ability to induce the cleavage of TSAP6. Because TSAP6/STEAP3 is a member of STEAP family, we determined whether other members of the family (STEAP1 and STEAP4) could be cleaved when RHBDD1 was co-expressed. Because some of the proteins contained N-terminal signal peptides, rendering N-terminal tags undetectable, all of the STEAP members were subcloned into a p3xFlag-CMV-14 construct containing a C-terminal 3xFlag tag. As shown in Fig. 1F, no cleavage bands were detected when STEAP1 and STEAP4 were co-expressed with RHBDD1.

Taken together, these results suggest that RHBDD1 could specifically induce the cleavage of TSAP6, indicating that TSAP6 is a potential target of RHBDD1-mediated proteolysis.

### RHBDD1-induced cleavage of TSAP6 occurs at multiple sites

**Figure 2:**
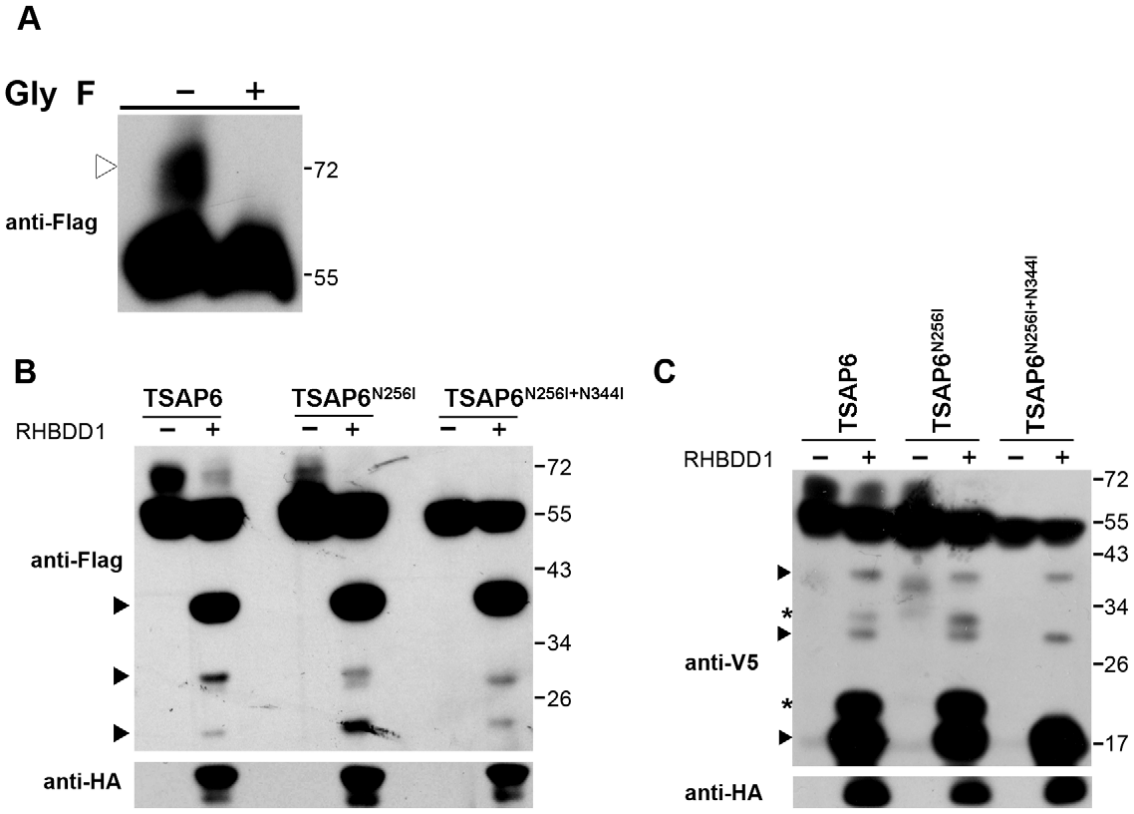
RHBDD1-induced TSAP6 cleavage occurs at multiple sites. A. Glycopeptidase F treatment of TSAP6. HEK-293T cell lysates transfected with TSAP6-Flag were treated with or without glycopeptidase F for 20 h at 37°C under denaturing conditions and blotted using an anti-Flag antibody. The posttranscripitional modified form of TSAP6 faded off (white arrowhead) after Glycopeptidase F treatment. B. RHBDD-mediated TSAP6 cleavage was not found to be dependent on the glycosylation of TSAP6. TSAP6 or its glycosylation site-mutated forms, TSAP6^N256I^ and TSAP6^N256I+N344I^, were co-transfected with control vector or a RHBDD1-HA-expressing vector in HEK-293T cells. At 36 h after transfection, the cells were lysed and immunoblotted using anti-Flag and anti-HA antibodies. The cleaved fragments of TSAP6 are indicated with black arrowheads. C.Immunodetection of RHBDD1-cleaved C-terminal fragments of TSAP6. RHBDD1-mediated TSAP6 cleavage generated three major fragments (black arrowheads). Two other fragments indicated with asterisks were the glycosylated form because they disappeared in the TSAP6^N256I+N344I^ construct.

Immunoblotting showed TSAP6 to be a 55–70 kDa doublet. This might be a result of posttranscriptional glycosylation. The posttranscriptional modification of TSAP6 hinders the mapping of TSAP6 cleavage sites, and is probably involved in the regulation of TSAP6 cleavage. In order to map the cleavage sites of TSAP6 and determine whether the posttranscriptional modification is involved in the regulation of TSAP6 cleavage, the type of posttranscriptional modification of TSAP6 first had to be examined. The 70 kDa band seemed to result from N-glycosylation because it faded off when treated with a nonspecific N-glycosidase, glycopeptidase F (Fig. 2A). Two potential glycosylation sites, Asn256 and Asn344, were predicted according to the NetNGlyc 1.0 server (www.cbs.dtu.dk/services/NetNGlyc/). To determine whether these two sites were the glycosylation sites of TSAP6, both of the Asn residues were serially mutated to Ile, and the ability of RHBDD1 to mediate cleavage of these mutant proteins was determined (Fig. 2B). The TSAP6^N256I^ mutation significantly reduced the glycosylated form of TSAP6, and the TSAP6^N256I+N344I^ double mutation completely abolished the glycosylated band. This confirmed that the 70 kDa band resulted from glycosylation. However, when RHBDD1 was co-transfected, the TSAP6 mutant proteins were cleaved normally, indicating that the cleavage did not rely on the N-glycosylation of TSAP6.

TSAP6 is a member of the oxydoreductase family, and it contains six transmembrane domains [15]. The total transmembrane domains of TSAP6 were predicted with SMART software (smart.embl-heidelberg.de/) ( Table 1).

**Table 1:** Predicted transmembrane domains of TSAP6 using SMART software.

Predicted transmembrane domains		
Number	Begin	End
TM1	209	231
TM2	258	280
TM3	305	327
TM4	359	381
TM5	394	416
TM6	431	453

To locate the cleavage regions of TSAP6, the samples in Fig. 2B were immunoblotted using an anti-V5 antibody to determine the C-terminal cleavage fragments of TSAP6. When RHBDD1 was co-transfected, three major C-terminal fragments were detected. They were approximately 38 kDa, 28 kDa, and 18 kDa in mass (Fig. 2C). We have shown that there were also three N-terminal cleavage fragments of TSAP6 detected. They were of approximately 38 kDa, 30 kDa and 22 kDa (Figs. 1A, 2B). These cleaved fragments were found to be relevant and added up to approximately the molecular weight of the full-length TSAP6 when the N-terminal and C-terminal fragments were added in pairs. Thus, we proposed that these fragments are cleaved fragments within three different regions. The major cleavage site is near the C-terminal one, generating an N-terminal 38-kDa cleaved fragment. This site was chosen for investigation of the mechanism of cleavage.

### TSAP6 is mainly cleaved around the C-terminal of its TM3 domain

**Figure 3:**
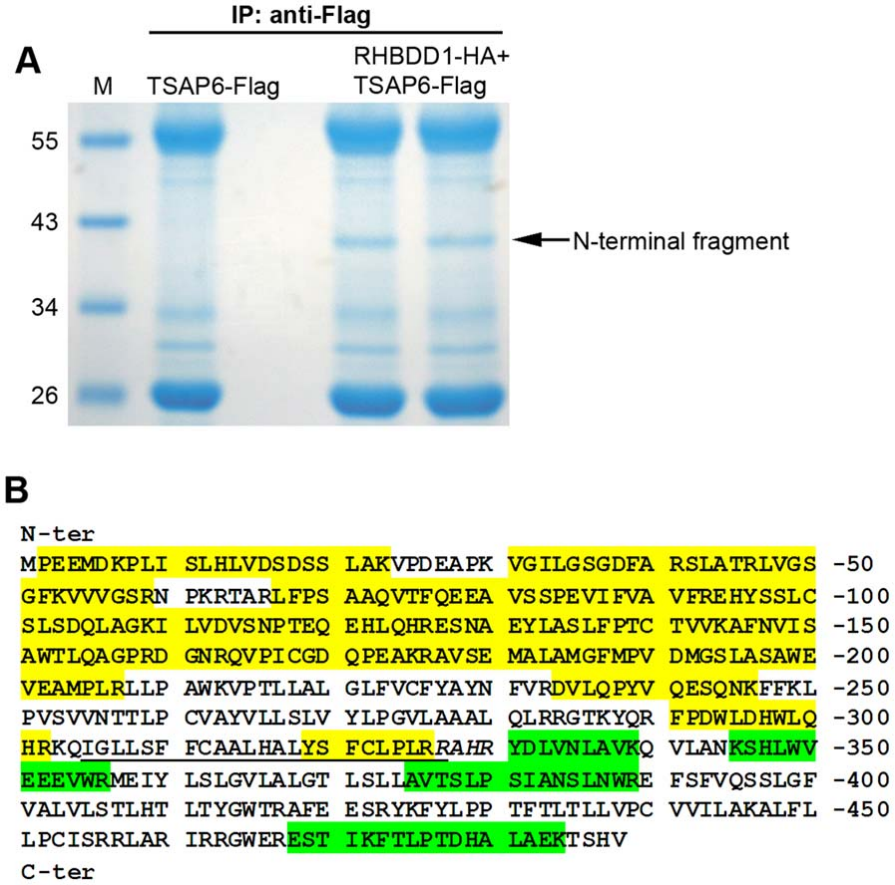
Determination of the major cleavage site within TSAP6 through mass spectrometric analysis. A. Immunoprecipitation of TSAP6 N-terminal fragments. The cell lysate of HEK-293T cells transfected with pcDNA6-Flag-TSAP6 and empty vector or RHBDD1-HA was immunoprecipitated with anti-Flag agarose. The immunoprecipitates were separated with SDS-PAGE and stained with Coomassie blue R-250. A 38-kDa protein band was observed when RHBDD1 was co-expressed. B. Mass spectrometric analysis of the resulting peptides. The TM3 domain of TSAP6 is underlined. The identified N-terminal peptides are marked in yellow, and the peptides identified from C-terminal fragment are shown in green. The predicted cleavage region is shown in italics.

To determine the cleavage sites of TSAP6, mass spectrometric analysis was used. Flag-tagged N-terminal and C-terminal cleavage fragments were generated by co-expressing the pcDNA6-Flag-TSAP6 or p3xFlag-TSAP6 constructs with RHBDD1-HA in 293T cells. The fragments were then immunoprecipitated with anti-Flag agarose, separated with SDS-PAGE, and stained with Coomassie blue R-250 (N-terminal fragment, Fig. 3A; C-terminal fragment, not shown). The purified fragments were digested and subjected to mass spectrometric analysis. As shown in Fig. 3B, a series of N-terminal peptides (yellow) and C-terminal peptides (green) were identified, with a N-terminal peptide (YSFCLPLR) located in the C-terminal of the third transmembrane domain (TM3). One peptide (YDLVNLAVK) that was adjacent to the TM3 region was identified in C-terminal fragment. The resulting peptides indicated that a cleavage site might lie in the short sequence (RAHR) between the two peptides.

**Figure 4:**
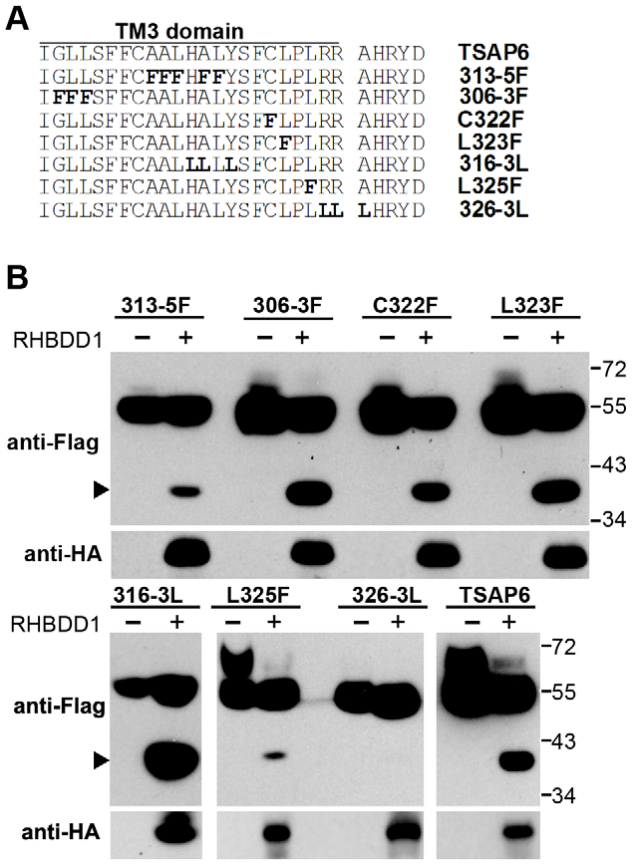
Location of the cleavage region of TSAP6 with mutagenic analysis. A. A series of mutations within and around TM3 was constructed. The mutated residues are shown in bold. B. The cleavage of TSAP6 and its mutant forms. The cleaved fragments are indicated with black arrowheads.

In order to confirm that the region contains a RHBDD1-induced cleavage site, we mutated a series of residues of TSAP6 into phenylalanine and leucine (Fig. 4A). We found that the cleavage was dramatically reduced when residues around the C-terminal region of TM3 were mutated (L325F and 326-3L mutations, Fig. 4B). However, mutations in other regions did not exert such a significant influence. L325F mutation significantly reduced the cleavage, while cleavage with 326-3L mutant protein was barely detectable, suggesting that the region around the C-terminal of the TSAP6 TM3 domain possesses a RHBDD1-induced cleavage site.

### The inactivation of endogenous RHBDD1 increases TSAP6-mediated exosome secretion

TSAP6 has been reported to regulate the nonclassical exosomal secretion pathway [17]. Because RHBDD1 induces cleavage in TSAP6 in cells, we evaluated the involvement of RHBDD1 in the TSAP6-mediated nonclassical secretion pathway.

**Figure 5:**
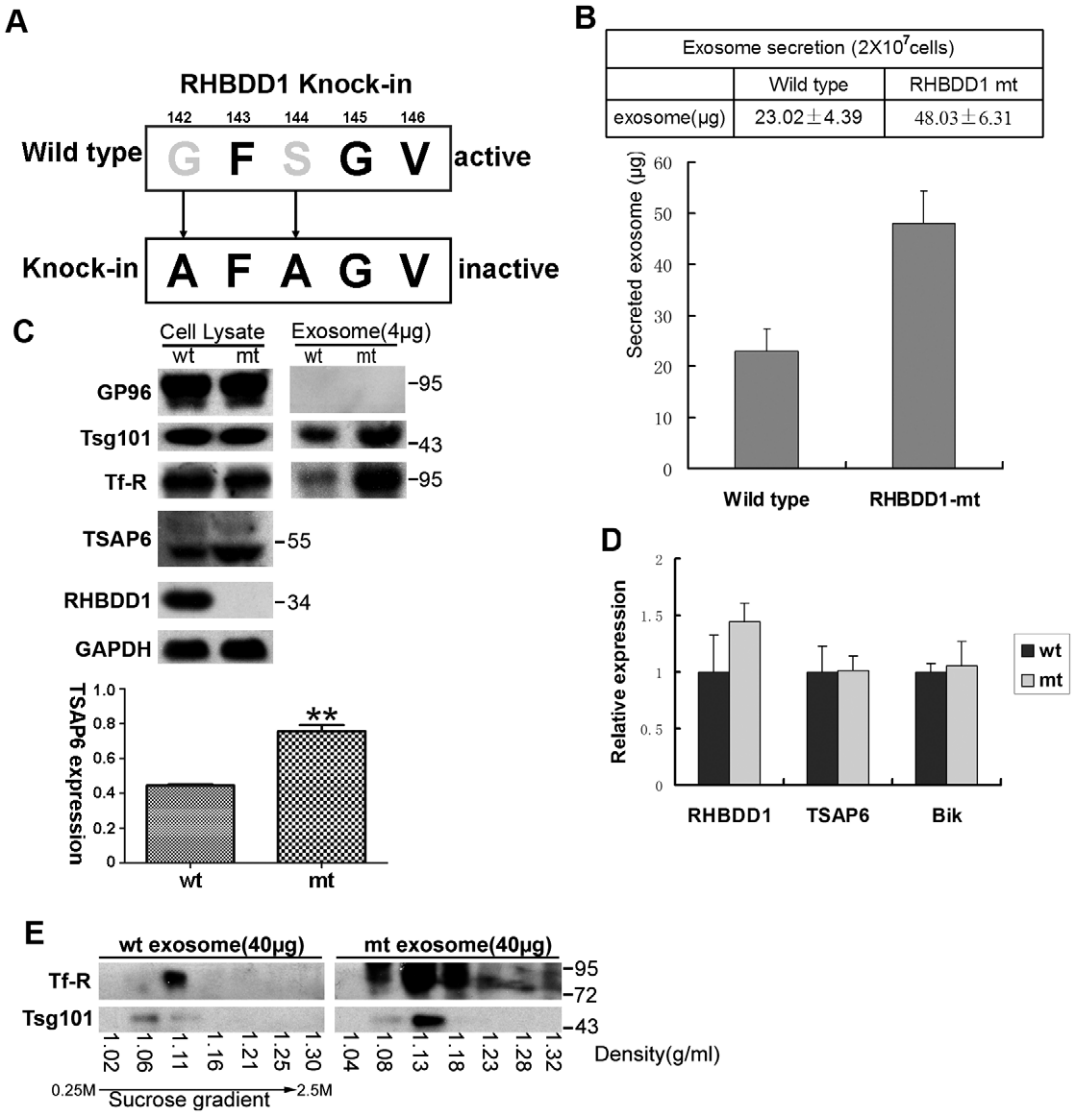
Analysis of exosome secretion in wild-type and RHBDD1-mt HCT116 cells. A. A schematic diagram of the targeting motif of RHBDD1 knock-in. B. Quantitative analysis of total exosomes secreted from 2×10^7^ HCT116 cells for 16 h. C. Immunodetection of Tsg101 and Tf-R contents in 4 µg of exosomal proteins from wild-type and RHBDD1-mt HCT116 cells. TSAP6 expression was normalized with GAPDH and shown below (** P<0.01). D. Real-time analysis of the relative mRNA levels of RHBDD1, TSAP6 and Bik in wild-type and RHBDD1-mt HCT116 cells. E. Western blot analysis of Tsg101 and Tf-R proteins on fractions collected from 40 µg of total exosome samples from wt and RHBDD1-mt HCT116 cells processed on a 0.25–2.5 M sucrose gradient.

Recently, a recombinant-adeno-associated virus-based homologous recombination-mediated somatic cell knock-in approach was developed to introduce various kinds of alterations to endogenous loci [36]. RHBDD1 possesses a GFSGV motif in its rhomboid domain, and this motif is critical to its enzymatic activity [32]. For this reason, we selected the knock-in approach to introduce two point mutations, G142A and S144A, into the endogenous loci of RHBDD1 in HCT116 colon cancer cells (Fig. 5A). After two rounds of targeting, HCT116 cells with mutant version of both alleles (RHBDD1-mt) were generated. They were verified using PCR analysis and DNA sequencing (data not shown). The total mRNA in HCT116-mt cells was extracted and reverse-transcribed into cDNA. Then, the cDNA encoding RHBDD1 was amplified and sequenced. This confirmed the mutations (Figure. S1). When immunoblotted with an anti-RHBDD1 antibody, the RHBDD1 protein was found to be barely detectable in RHBDD1-mt cells (Fig. 5C). This low level of expression was probably the result of rapid proteasomal degradation, as indicated by the fact that the proteasome inhibitor MG132 partially restored RHBDD1 in the mutated cells (data not shown). To investigate endogenous TSAP6, we generated a monoclonal anti-TSAP6 antibody. However, the antibody was shown to be rather poor in quality and there were non-specific bands in the blot, despite that it did recognize bands corresponding to TSAP6 (Figure S2).

As shown in Fig. 5C, the level of TSAP6 was found to be significantly increased in RHBDD1-mt cells, which was probably the result of reduced RHBDD1-mediated proteolysis of endogenous TSAP6. Because the change in the level of TSAP6 could also have resulted from increased gene transcription, the relative mRNA level of TSAP6 was quantified using RT-PCR. No significant differences were found (Fig. 5D). Similar results were also observed with Bik, and the mRNA of RHBDD1 was found to be slightly elevated in RHBDD1-mt cells. In this way, abrogation of RHBDD1-mediated proteolysis seemed to be responsible for the increase in TSAP6 protein level in RHBDD1-mt HCT116 cells.

Next, we analyzed whether RHBDD1 inactivation had an influence on TSAP6-mediated exosome secretion. According to a published protocol for exosome isolation and characterization, we isolated the secreted exosomes in wild-type and RHBDD1-mt HCT116 cells through differential ultracentrifugation approach [37]. The secreted exosome proteins were quantified and found to be significantly elevated when RHBDD1 was mutated (Fig. 5B). The experiment was repeated at least four times, and similar results were obtained. Because some non-specific contaminations might be associated with exosomes purified by differential ultracentrifugation, we further detected two exosomal components, tumor susceptibility gene 101 (Tsg101) and transferrin receptor (Tf-R) to determine whether there was an increase of exosome components in the samples. Gp96 (glycoprotein 96) was used as a control to determine whether there was any contamination with cellular debris in exosome samples (Fig. 5C). The exosomal components Tsg101 and Tf-R were found to be significantly elevated in exosome samples from RHBDD1-mt cells, indicating that RHBDD1 inactivation could significantly increase the quantity of total secreted exosomes. Besides, the isolated exosome samples were evaluated through sucrose gradient ultracentrifugation, which is an accurate method to define the exosomal fraction [37]. Both of the exosomal components were found to accumulate mostly in the 1.08–1.23 g/ml fractions, indicating that the components are located in exosome fractions (Fig. 5E).

**Figure 6:**
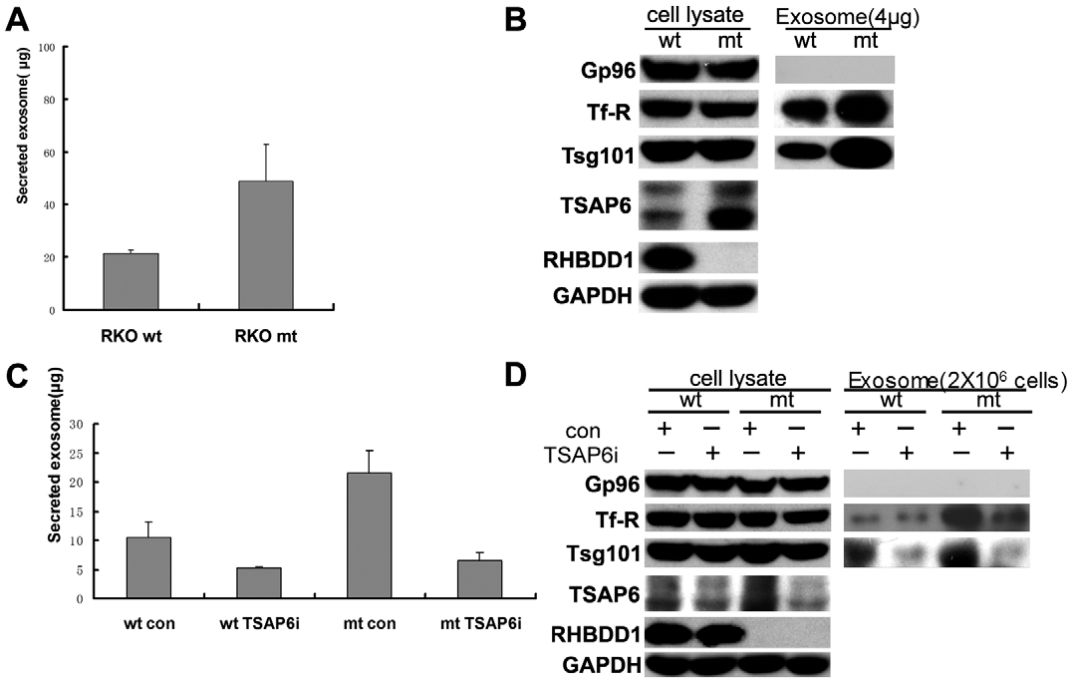
Exosome secretion in RKO cells and TSAP6 dependency of RHBDD1's role in exosome secretion. A. Total exosome proteins secreted from 2×10^7^ wild-type (RKO wt) and RHBDD1-mt (RKO mt) RKO cells in 16 h. B. Immunodetection of exosome components and cellular TSAP6 in wt and mt RKO cells. C. Wild-type and RHBDD1-mt HCT116 cells infected with control shRNA (con) or TSAP6 shRNA (TSAP6i) lentivirus were plated and assayed for exosome secretion. Total exosomes secreted from 1×10^7^ HCT116 cells were measured. D. Western blot analysis of cell lysates and exosome samples. Cell lysates or exosome samples secreted from 2×10^6^ cells were seperated with SDS-PAGE and blotted with indicated antibodies.

In order to determine whether elevated exosome secretion caused by RHBDD1 inactivation was reproducible in other types of cells, we introduced the same mutations into endogenous RHBDD1 in RKO colon cancer cells. Exosome secretion was assayed in wild-type (RKO wt) and RHBDD1-mt (RKO mt) cells (Fig. 6A). The exosome components, Tsg101 and Tf-R, were also detected in equal amount of exosome samples (Fig. 6B). Both of total secreted exosomes and the exosomal components were elevated significantly when RHBDD1 was mutated. These findings indicated that RHBDD1 inactivation in HCT116 and RKO cells had very similar influence on exosome secretion. In this way, RHBDD1 mutation was shown to be associated with elevated exosome secretion, indicating that RHBDD1 might have a role in the regulation of exosome secretion.

It has been documented that TSAP6 played a role in regulating exosome secretion. Thus, we speculated that RHBDD1 might facilitate the regulation of exosome secretion through modulating the level of TSAP6 in cells. In order to determine whether the elevated exosome secretion in RHBDD1 mutant cells was TSAP6-dependent, we knocked down the expression of TSAP6 in wild-type and RHBDD1-mt HCT116 cells, and assayed exosome secretion in these cells. Exosome secretion was found to be significantly decreased in both wild type and RHBDD1-mt HCT116 cells when TSAP6 was knocked down (Fig. 6C). More importantly, the ratio of secreted exosomes between RHBDD1-mt and wild-type cells was reduced dramatically when TSAP6 was knocked down, from 2.05 (21.6 µg/10.5 µg) to 1.25 (5.5 µg/4.4 µg), which is consistent with the reduced difference in TSAP6 expression. The differential levels of exosomal components in wild type and RHBDD1-mt cells were also reduced significantly when TSAP6 was knocked down (Fig. 6D). These findings indicated that the elevation of exosome secretion by RHBDD1 inactivation was very likely to be TSAP6-dependent.

### RHBDD1 inactivation promotes exosome-induced apoptosis in Jurkat cell

Recent studies have highlighted exosomes as a multifunctional organelle with biological significance. Exosomes have been found to participate in cell-cell communication, tumor progression and immune regulation through specialized exosomal components. Recently, FasL and Trail have been identified as exosomal components secreted from cancer cells. They can cause apoptosis in T lymphocytes through the activation of their corresponding receptors [24]. For this reason, we determined whether exosomes secreted from HCT116 cells also contained these ligands and whether exosomes secreted from RHBDD1-mt cells had enhanced biological activity relative to wt HCT116 cells. Jurkat cells were used as the model for T lymphocytes in this assay [24].

**Figure 7:**
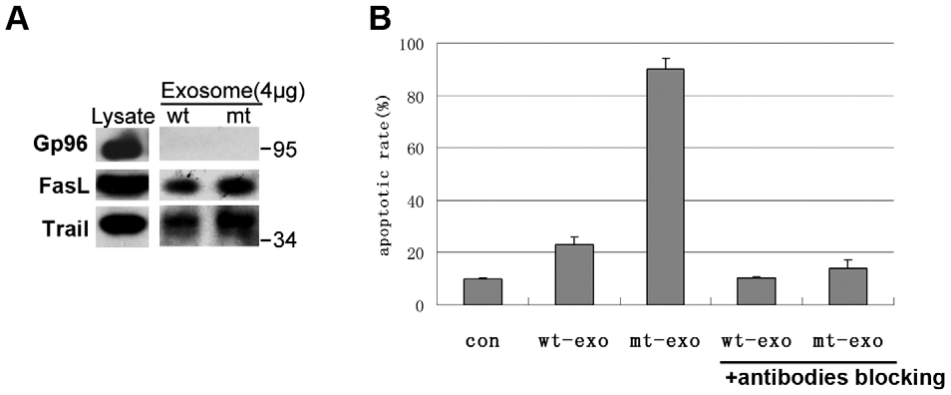
Exosome samples secreted from RHBDD1-mt cells showed elevated ability to induce Jurkat cell apopotosis. A. Immunodetection of FasL and Trail in 4 µg of exosomal proteins secreted from wild-type and RHBDD1-mt HCT116 cells. B. The apoptotic rate of Jurkat cells after incubation with exosome samples purified from wild-type or RHBDD1-mt HCT116 cells.

As shown in Fig. 7A, FasL and Trail were both detected in the exosome samples. In addition, levels of both ligands were increased in exosomes secreted from RHBDD1-mt cells. Furthermore, the exosomes from RHBDD1-mt cells had significantly enhanced ability to induce apoptosis in Jurkat cells. This ability appeared to be primarily dependent on FasL and Trail because FasL and Trail antibodies dramatically attenuated the apoptotic effect of exosomes (Fig. 7B). Taken together, these results show that RHBDD1 has influences on the secretion of the exosomal components FasL and Trail, which partly contribute to the role of exosomes in inducing the apoptosis in Jurkat cells.

## Discussion

**Figure 8:**
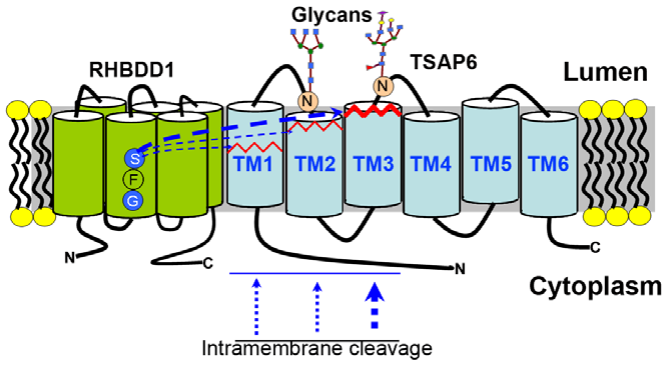
A hypothetical schematic diagram of RHBDD1 mediated TSAP6 proteolysis.

It has been a decade since rhomboid proteases were identified, and great progress has been made since the discovery of this intriguing intramembrane protease family [38]. In the present study, we show that TSAP6 was cleaved specifically in response to RHBDD1 overexpression. Further, we identified a cleavage site in the C-terminal region of TSAP6 TM3 domain, which resembles an intramembrane cleavage site. Finally, endogenous TSAP6 became enriched when RHBDD1 was inactivated, indicating that RHBDD1 could restrict the level of endogenous TSAP6 protein in cells. Taken together, these results indicate that TSAP6 is a target of RHBDD1-induced proteolysis. Besides, TSAP6 was identified through yeast two-hybrid assay with RHBDD1 as bait, implicating that TSAP6 might have direct binding with RHBDD1 and was very probably catalyzed directly by the protease (Fig. 8). However, the substrate-recognition-mechanisms of rhomboid proteases are diverse and complicated and the model for how RHBDD1 recognizes and cleaves its substrates has not yet been proposed. Thus, whether TSAP6 is directly cleaved by RHBDD1 remains to be a question of doubt and needs to be verified when there is better understanding for the protease.

To data, all well-confirmed rhomboid substrates are type I membrane proteins. However, evidences indicated that other types of transmembrane proteins might also be the substrates of rhomboid proteases. Type II membrane protein Star is cleaved in transmembrane region in response to Rhomboid-1 overexpression [39]. GlpG was shown to cleave synthetic multi-pass membrane proteins [40]. In this study, intact multi-pass membrane protein was found to might also be cleaved by rhomboids. Intriguingly, both of the proposed multi-pass membrane proteins seemed to be cleaved in type II orientation. These findings indicated that rhomboid proteases might have a broader substrate profile than previously supposed.

We found that RHBDD1 inactivation enriched endogenous TSAP6 and increased exosome secretion in cells. Because it has been established that TSAP6 promotes exosome secretion, it is highly possible that the role for RHBDD1 in regulating exosome secretion mainly involves restricting TSAP6 [16], [17]. By knocking down the endogenous TSAP6, the increase in exosome secretion induced by RHBDD1 inactivation was reduced. This was consistent with the idea that TSAP6 is a possible intermediate for RHBDD1's regulatory role in exosome secretion.

A large percentage of rhomboid proteases are involved in the cellular secretion pathway. Our findings indicate that RHBDD1 is involved in the regulation of exosomal secretion probably through modulating the amount of TSAP6. This might play a role in inducing apoptosis in T lymphocyte. In this respect, the exosomes secreted from cancer cells might contribute to immune suppression and cancer progression, as previously reported [41], [42]. However, an increasing number of studies have indicated that exosomes are multi-functional organelles with biological significance. The biological functions of exosomes are complicated and depend on various factors, including the numerous components of the exosomes, the cells from which they originate, and the cells with which they interact. For example, exosomes originating from immune cells, such as dendritic cells and B cells, have been found to play a role in regulating antigen presentation, which might promote immune systems to target cancer cells [43], [44]. In this way, the biological consequence of RHBDD1 in affecting exosome secretion remains unclear. More work must be performed to determine with certainty the biological significance of exosome secretion and how RHBDD1 plays a role in this regard.

## Materials and Methods

### Ethics statement

The Animal Ethics Committee of National Research Institute for Family Planning Beijing approved the animal experimentation protocols. All animal experiments were performed in accordance with the Guidelines for the Care and Use of Laboratory Animals as established by the Chinese Council on Animal Care. All the protocol numbers we received from the Animal Ethicics Committee of National Research Institute for Family Planning are as follows: 20090316, 20091016 and 20100505.

### Cell Culture, cDNAs, and Transfection

HEK-293T (Cell Resource Center, PUMC) cells were cultured in Dulbecco's modified Eagle's medium (Invitrogen) supplemented with 10% fetal calf serum. HCT116 (Cell Resource Center, PUMC) and RKO (Cell Resource Center, PUMC) cells were cultured in Iscove's modified Dulbecco's medium (Invitrogen) supplemented with 10% fetal calf serum (Invitrogen). The 293T cells were transfected using Entranster D (Engreen Biosystem Co, Ltd.). Jurkat cells (Clone E6-1, Cell Resource Center, PUMC) were cultured in RPMI-1640 medium supplemented with 10% fetal calf serum (Hyclone).

The cDNA encoding TSAP6 was amplified from a 293T cDNA library using PCR using the following primers: sense, 5′-ATCAAGCTTACCATGCCAGAAGAGATGGAC-3′, and anti-sense, 5′-ATCCTCGAGGTGACCGTACGTGGCTCGTCTTCTCGGCC-3′. A Flag tag (DYKDDDDK) was added in the NheI and HindIII sites of the pcDNA6-V5/His-B plasmid (Invitrogen). The TSAP6 PCR product was subcloned into the HindIII and XhoI sites of the pcDNA6-Flag/V5 construct. The TSAP6 mutations were constructed using overlapping extension PCR. RHBDD1 and its S144A mutation constructs were described previously [32]. Myc-tagged RHBDD1- and RHBDL2-expressing vectors were constructed by inserting the coding sequences into the HindIII and XhoI sites of pcDNA6-Myc/His-B (Invitrogen). The C-terminal 3xFlag-tagged STEAP1, STEAP4 and TSAP6 constructs were generated by inserting the coding sequences into the HindIII and XbaI sites of p3xFlag-CMV-14 (Sigma).

### Antibodies, Co-immunoprecipitation, and Immunoblotting

The following antibodies were purchased: rabbit polyclonal anti-RHBDD1 (HPA013972, Sigma), Flag (F3165, Sigma), Myc (sc-40, Santa Cruz), HA (H3663, Sigma), GAPDH (TA-08, ZSbio), Gp96 (bs-0194R, Bios Biotech), Tsg101(bs-1365R, Bios Biotech), Tf-R (sc-32272, Santa Cruz), FasL (#4273, Cell Signaling, for western blot analysis), FasL (556371, BD Biosciences, for antibody-blocking assays), Trail (550515, BD Biosciences), and HRP-conjugated goat anti-mouse and goat anti-rabbit antibodies (ZSbio). The monoclonal anti-TSAP6 antibody was generated through the immunization of a BALB/c mouse (Laboratory Animal Centre of PUMC, Beijing) with a 6×His-tagged purified recombinant protein corresponding to the N-terminal 1-126-amino acid fragment of human TSAP6. Hybridomas were screened by an indirect enzyme-linked immunosorbent assay (ELISA) using the 6×His-tagged recombinant protein. Immunoblotting assays were performed as described previously [32].

### Immunoprecipitation and mass spectrometric analysis

5×10^6^ 293T cells transfected with 8 µg RHBDD1-HA and 8 µg pcDNA6-Flag-TSAP6 or p3xFlag-TSAP6 plasmids were lysed in lysis buffer (20 mM Tris, pH 7.5, 200 mM NaCl, 1% NP-40, 10% glycerol, 1 mM DTT and completed protease inhibitor cocktail (Roche)). They were then immunoprecipitated with 40 µl anti-Flag slurry ( A2220, Sigma). The immunoprecipitates were separated with 10% SDS-PAGE and stained with Coomassie blue R-250.

The protein bands were cut and used for in-gel digestion with trypsin. The resulting peptides were analyzed with LTQ Orbitrap Velos mass spectrometer (Thermo Scientific).

### RNA extraction and relative quantitative real-time PCR

RNA was extracted from HCT116 cells using TRIzol reagent (Invitrogen) according to the manufacturer's instructions. The total RNA was reverse-transcribed into cDNA using ReverTra Ace-α (Toyobo). Relative quantitative RT-PCR was performed using the TaqMan Universal PCR Master Mix (Applied Biosystems) on an iCycler IQ5 PCR machine (Bio-Rad). The primers for RT-PCR were as follow: RHBDD1: sense, 5′-CTCTGGGACCGAGGAAATACC-3′, and anti-sense, 5′-ACCTCACTGGCTATCGAATCTGT-3′.;TSAP6: sense, 5′-TGCAAACTCGCTCAACTGGAG-3′, and anti-sense, 5′-GAAGGTGGGAGGCAGGTAGAA-3′; Bik: sense, 5′-ACCATGGAGGTTCTTGGCA-3′, and anti-sense, 5′-AGGCTCACGTCCATCTCGTC-3′; Actin: sense, 5′-AGGCCAACCGCGAGAAGAT-3′, and anti-sense, 5′-TCACCGGAGTCCATCACGAT-3′.

### Exosome secretion and isolation

Exosome isolation and characterization assays were carried out in accordance with a published protocol [37]. For the exosome secretion assay, 2×10^7^ HCT116 or RKO cells (1×10^7^ cells were used in TSAP6 knockdown assay) were plated and cultured for 24 h. The cells were incubated with exosome-free IMDM medium, which was prepared using ultracentrifugation at 100,000 g for 16 h at 4°C. After 16 h of incubation, the medium was collected and centrifuged twice at 300 g for 5 min to remove cell debris. The medium was further centrifuged at 12,000 g for 30 min and filtered through a 0.22 µm filter. Exosome pellets were isolated using centrifugation at 100,000 g for 2 h at 4°C in a Beckman L-100 XP Ultracentrifuge. The isolated exosomes were washed with a large volume of PBS and resuspended in 100 µl PBS. The amount of exosomes secreted was quantified using a BCA protein assay kit (Thermo Scientific). The sucrose gradient used to purify exosomes was similar to a method described previously [17]. Briefly, 40 µg of total exosomes was centrifuged at 100,000 g for 16 h in 2.2 ml of a 0.25 M–2.5 M sucrose gradient at 4°C in a Beckman LT-100 Ultracentrifuge. Each fraction (300 µl) was added with 75 µl of 5× SDS sample buffer, boiled for 5 min and analyzed using western blotting.

### Deglycosylation assay

The treatment of TSAP6 with glycopeptidase F (Takara Biotech) was performed under denaturing conditions according to the manufacturer's protocol.

### Exosome-induced apoptosis assay

1×105 Jurkat cells were incubated with 5 µg of purified exosome samples for 60 h, stained with annexin-V-FITC/PI (Neobioscience) and analyzed using an Accuri C6 flow cytometer (BD Biosciences). For antibody-blocking assays, the exosome samples were pre-incubated with 1 µg/ml anti-FasL and anti-Trail antibodies for 30 min.

### TSAP6 RNAi assay

Lentivirus human TSAP6-targetting oligonucleotides were synthesized and packaged by GENECHEM Inc. The TSAP6 targeting sequences was: 5′- GAGGGAGTTCAGCTTCGTTCA -3′. HCT116 colon cancer cells were infected with TSAP6-shRNA-lentivirus or control shRNA virus. At 72 h after infection, the cells were subjected to exosome secretion assay.

## Supporting Information

Figure S1 **Alignment of knock-in cDNA and the coding sequence of RHBDD1.** Note that two mutations were detected in the knock-in cDNA of RHBDD1, which codes an inactivated form of RHBDD1.
(DOC)

Figure S2 **Verification of TSAP6 antibody with cell lysates.** Protein samples were seperated with SDS-PAGE and blotted with monoclonal anti-TSAP6 antibody. Line 1, HCT116 cell lysate. Line 2, 293T cell lysate transfected with TSAP6-Flag.
(TIF)
