# Supplementary material for: Exosome-Related Multi-Pass Transmembrane Protein TSAP6 Is a Target of Rhomboid Protease RHBDD1-Induced Proteolysis
Source: PLoS One. 2012 May 18;7(5):e37452. doi: 10.1371/journal.pone.0037452 (PMC3356283; doi:10.1371/journal.pone.0037452)
Supplement: Figure S1 — Alignment of knock-in cDNA and the coding sequence of RHBDD1. Note that two mutations were detected in the knock-in cDNA of RHBDD1, which codes an inactivated form of RHBDD1. (DOC) [file pone.0037452.s001.doc]

Upper line：RHBDD1 knock-in cDNA

Lower line：the coding sequence of RHBDD1

36 ATGCAACGGAGATCAAGAGGGATAAATACTGGACTTATTCTACTCCTTTCTCAAATCTTC

1 ATGCAACGGAGATCAAGAGGGATAAATACTGGACTTATTCTACTCCTTTCTCAAATCTTC

96 CATGTTGGGATCAACAATATTCCACCTGTCACCCTAGCAACTTTGGCCCTCAACATCTGG

61 CATGTTGGGATCAACAATATTCCACCTGTCACCCTAGCAACTTTGGCCCTCAACATCTGG

156 TTCTTCTTGAACCCTCAGAAGCCACTGTATAGCTCCTGCCTTAGTGTGGAGAAGTGTTAC

121 TTCTTCTTGAACCCTCAGAAGCCACTGTATAGCTCCTGCCTTAGTGTGGAGAAGTGTTAC

216 CAGCAAAAAGACTGGCAGCGTTTACTGCTCTCTCCCCTTCACCATGCTGATGATTGGCAT

181 CAGCAAAAAGACTGGCAGCGTTTACTGCTCTCTCCCCTTCACCATGCTGATGATTGGCAT

276 TTGTATTTCAATATGGCATCCATGCTCTGGAAAGGAATAAATCTAGAAAGAAGACTGGGA

241 TTGTATTTCAATATGGCATCCATGCTCTGGAAAGGAATAAATCTAGAAAGAAGACTGGGA

336 AGTAGATGGTTTGCCTATGTTATCACCGCATTTTCTGTACTTACTGGAGTGGTATACCTG

301 AGTAGATGGTTTGCCTATGTTATCACCGCATTTTCTGTACTTACTGGAGTGGTATACCTG

396 CTCTTGCAATTTGCTGTTGCCGAATTTATGGATGAACCTGACTTCAAAAGGAGCTGTGCT

361 CTCTTGCAATTTGCTGTTGCCGAATTTATGGATGAACCTGACTTCAAAAGGAGCTGTGCT

**A A**

456 GTAGCTTTCGCAGGAGTTTTGTTTGCTTTGAAAGTTCTTAACAACCATTATTGCCCTGGA

| |

421 GTAGGTTTCTCAGGAGTTTTGTTTGCTTTGAAAGTTCTTAACAACCATTATTGCCCTGGA

**G S**

516 GGCTTTGTCAACATTTTGGGCTTTCCTGTACCGAACAGATTTGCTTGTTGGGTCGAACTT

481 GGCTTTGTCAACATTTTGGGCTTTCCTGTACCGAACAGATTTGCTTGTTGGGTCGAACTT

576 GTGGCTATTCATTTATTCTCACCAGGGACTTCCTTCGCTGGGCATCTGGCTGGGATTCTT

541 GTGGCTATTCATTTATTCTCACCAGGGACTTCCTTCGCTGGGCATCTGGCTGGGATTCTT

636 GTTGGACTAATGTACACTCAAGGGCCTCTGAAGAAAATCATGGAAGCATGTGCAGGCGGT

601 GTTGGACTAATGTACACTCAAGGGCCTCTGAAGAAAATCATGGAAGCATGTGCAGGCGGT

696 TTTTCCTCCAGTGTTGGTTACCCAGGACGGCAATACTACTTTAATAGTTCAGGCAGCTCT

661 TTTTCCTCCAGTGTTGGTTACCCAGGACGGCAATACTACTTTAATAGTTCAGGCAGCTCT

756 GGATATCAGGATTATTATCCGCATGGCAGGCCAGATCACTATGAAGAAGCACCCAGGAAC

721 GGATATCAGGATTATTATCCGCATGGCAGGCCAGATCACTATGAAGAAGCACCCAGGAAC

816 TATGACACGTACACAGCAGGACTGAGTGAAGAAGAACAGCTCGAGAGAGCATTACAAGCC

781 TATGACACGTACACAGCAGGACTGAGTGAAGAAGAACAGCTCGAGAGAGCATTACAAGCC

876 AGCCTCTGGGACCGAGGAAATACCAGAAATAGCCCACCACCCTACGGGTTTCATCTCTCA

841 AGCCTCTGGGACCGAGGAAATACCAGAAATAGCCCACCACCCTACGGGTTTCATCTCTCA

936 CCAGAAGAAATGAGGAGACAGCGGG

901 CCAGAAGAAATGAGGAGACAGCGGC

Figure S1
