# Supplementary figures and images for: Exosome-Related Multi-Pass Transmembrane Protein TSAP6 Is a Target of Rhomboid Protease RHBDD1-Induced Proteolysis
Source: PLoS One. 2012 May 18;7(5):e37452. doi: 10.1371/journal.pone.0037452 (PMC3356283; doi:10.1371/journal.pone.0037452)

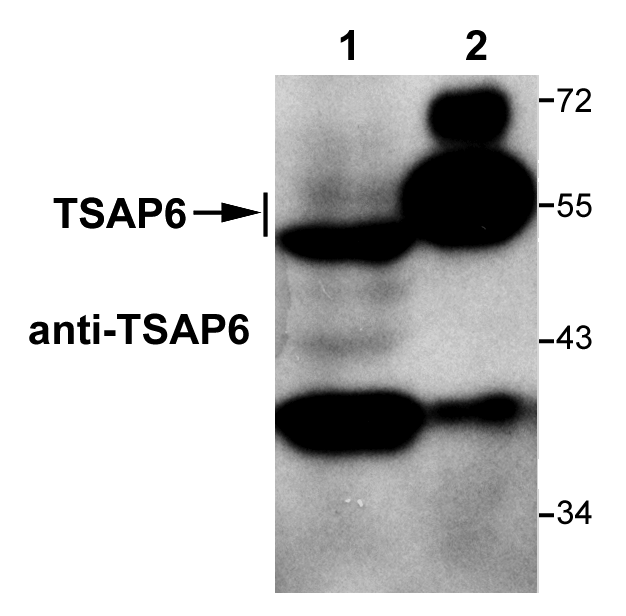

Supplement: Figure S2 — Verification of TSAP6 antibody with cell lysates. Protein samples were seperated with SDS-PAGE and blotted with monoclonal anti-TSAP6 antibody. Line 1, HCT116 cell lysate. Line 2, 293T cell lysate transfected with TSAP6-Flag. (TIF) [file pone.0037452.s002.tif]
